# Laparoscopic ventral hernia repair in patients with obesity: should we be scared of body mass index?

**DOI:** 10.1007/s00464-021-08489-9

**Published:** 2021-05-04

**Authors:** Marianna Maspero, Camillo Leonardo Bertoglio, Lorenzo Morini, Bruno Alampi, Michele Mazzola, Valerio Girardi, Andrea Zironda, Gisella Barone, Carmelo Magistro, Giovanni Ferrari

**Affiliations:** 1 Division of Oncologic and Minimally Invasive General Surgery, Niguarda General Hospital, Milan, Italy; 2 Università degli Studi di Milano, Milan, Italy

**Keywords:** Ventral hernia, Laparoscopic ventral hernia repair, Obesity, Body mass index

## Abstract

**Background:**

Obesity is a risk factor for ventral hernia development and affects up to 60% of patients undergoing ventral hernia repair. It is also associated with a higher rate of surgical site occurrences and an increased risk of recurrence after ventral hernia repair, but data is lacking on the differences between obesity classes.

**Methods:**

Between 2008 and 2018, 322 patients with obesity underwent laparoscopic ventral hernia repair in our department: class I *n* = 231 (72%), II *n* = 55 (17%), III *n* = 36 (11%). We compared short and long-term outcomes between the three classes.

**Results:**

Patients with class III obesity had a longer median length of hospital stay compared to I and II (5 days versus 4 days in the other groups, *p* = 0.0006), but without differences in postoperative complications or surgical site occurrences. After a median follow up of 49 months, there were no significant differences in the incidence of seroma, recurrence, chronic pain, pseudorecurrence and port-site hernia. At multivariate analysis, risk factors for recurrence were presence of a lateral defect and previous hernia repair; risk factors for seroma were immunosuppression, defect > 15 cm and more than one previous hernia repair; the only risk factor for postoperative complications was chronic obstructive pulmonary disease.

**Conclusion:**

Class III obesity is associated with longer length of hospital stay after laparoscopic ventral hernia repair, but without differences in postoperative complications and long-term outcomes compared with class I and class II obesity.

Obesity is the main independent risk factor for the development of both primary and incisional ventral hernia (VH), with mechanisms related to augmented intra-abdominal pressure and delayed tissue repair [1, 2].

The prevalence of obesity has been rising continuously over the last decades. Nowadays, up to 60% of patients undergoing ventral hernia repair (VHR) presents with a body mass index (BMI) ≥ 30 and nearly 8% of patients undergoing bariatric surgery may have a concomitant VH [3].

The most appropriate timing and indication for VHR in patients with obesity is still under debate. Until the Nineties, VHR in obese patients was considered only after weight loss because of the high incidence of postoperative complications and recurrence rate. The introduction of laparoscopic repair led to a decrease in postoperative surgical complications compared to open VHR (3.4 vs 10.5%, *p* < 0.001) [3] in the general population, and encouraged the adoption of this technique also for patients with obesity [4–6].

LVHR in patients with obesity has many advantages compared to open VHR: it is associated with reduced overall complications and total hospital costs [7], a 70–80% reduction in surgical site infections (SSI) [8], less postoperative discomfort and a faster functional recovery. Laparoscopy also allows the identification of non-clinically evident hernia defects, a common occurrence in patients with obesity [9, 10]. For these reasons, LVHR should be preferred to open repair if the characteristics of the defect allow it [11, 12].

The impact of increasing BMI on postoperative outcomes following LVHR is still unclear, with limited scientific evidence based on small-sized case series. Some studies [6] have shown no differences between different obesity classes and postoperative outcomes following LVHR, while others have reported a higher recurrence rate in patients with class II [13] and class III obesity [14, 15]. A deeper understanding is pivotal for tailoring the best surgical approach for this group of patients.

The aim of our study was to investigate the correlation between obesity classes and postoperative outcomes in LVHR.

## Materials and methods

### Patient selection and study design

We retrospectively reviewed our prospectively collected institutional database on more than 800 LVHR performed at our Institution between 2008 and 2018, and selected patients with BMI ≥ 30 kg/m^2^. The selected patients were further divided according to their obesity class into three groups: obesity class I (OC1, with BMI ≥ 30 kg/m^2^ and < 35), obesity class II (OC2, with BMI ≥ 35 kg/m^2^ and < 40) and obesity class III (OC3, with BMI ≥ 40 kg/m^2^).

All patients were operated consecutively by abdominal wall surgeons or by surgeons-in-training under their tutoring. The surgical technique was standardized, and all patients received implantation of an expanded polytetrafluoroethylene (e-PTFE) mesh (Dual Mesh, W.L. Gore & Associates, Flagstaff, AZ, USA).

The study protocol followed the ethical guidelines of the 1975 Declaration of Helsinki (as revised in Brazil 2013). Formal approval from the Institutional Review Board was not deemed necessary due to the retrospective, observational and anonymous nature of the study. The results are reported according to Strengthening the Reporting of Observational Studies in Epidemiology (STROBE) [16].

The primary endpoint of this study was hernia recurrence. Secondary endpoints were short- and long-term complications.

### Patient management

During their first surgical outpatient visit, all patients were counseled on the importance of weight loss on short- and long-term surgical outcomes of LVHR, and offered time between the first counseling and the operation to achieve weight loss, unless particularly symptomatic.

All patients were operated consecutively by abdominal wall surgeons or by surgeons-in-training under their tutoring. The surgical technique was standardized, and all patients received implantation of an expanded polytetrafluoroethylene (e-PTFE) mesh (Dual Mesh, W.L. Gore & Associates, Flagstaff, AZ, USA).

Follow up was carried out with physical examination during outpatient visits in our clinic. Computed tomography was requested in case of clinical suspicion of recurrence or trocar-site hernia. Follow up visits were scheduled every six months for the first year, then yearly for the next four years. After five years, the follow up was considered completed.

### Data collection and statistical analysis

Data on demographic variables, characteristics of the defect, characteristics of the procedure and short- and long-term postoperative outcomes were systematically recorded on an electronic spreadsheet. Hernia defects were labeled according to the European Hernia Society (EHS) classification [17]. The defect area was calculated using the following formula

$${\text{defect area}} = \frac{{{\text{width }} \times {\text{ length}}}}{2}\;\, \times \,\;\pi$$

In case of multiple defects, the width was considered as the distance between the uppermost margin of the most cranial defect and the lower margin of the most caudal, as per EHS guidelines [17]. The same method was applied to the length, but on the horizontal axis. The defect area was then approximated using the same formula as for single defects.

The Clavien-Dindo classification [18] was used to grade short-term complications.

Statistical analysis was carried out with the commercially available software JMP®, Version 14 (SAS Institute Inc., Cary, NC, 1989–2019). Variables were compared using the one-way analysis of variance (ANOVA); p values of < 0.05 at ANOVA were further analysed with Tukey's test to assess significance between groups. Univariate regression analysis was performed to identify factors independently associated with 30-day postoperative complications, seroma and recurrence. Variables with p values < 0.10 at univariate analysis were further analysed with multivariate logistic regression analysis using Firth’s correction for rare events. All statistical tests were two-sided, and resultant p values of < 0.05 were considered statistically significant.

### Surgical technique

The patient is placed in a supine position. The pneumoperitoneum is induced with a Veress needle, usually in the left subcostal space. One 12 mm optical trocar and two 5 mm operative trocars are then placed. Trocar positioning depends on the size and location of the defect: care must be put in inserting the trocars in a way that will allow for mesh placement with at least 5 cm of overlap on all sides. Left-sided and midline defects are usually approached with the surgeon placed at the patient’s right, while a right-sided defect may be approached from the left.

The procedure begins with the exploration of the abdominal cavity and the identification of the defect. The hernia content is reduced and liberated from any adhesions with the hernia sac. The abdominal wall around the defect is then prepared for proper mesh implantation, ensuring there will be no fat between the mesh and the abdominal wall.

The defect is measured upon lowering the pneumoperitoneum to 8–10 mmHg. If the defect is small enough and the conditions of the abdominal wall tissues are such that its closure won’t exert an excessive tension on the abdominal wall, the margins of the defect may be approximated with a transparietal running suture. Defect closure provides a larger contact surface between the mesh and the abdominal wall for mesh implantation and potentially diminishes the rate of postoperative seroma development.

After identification of the most appropriate mesh size, the ePTFE mesh is introduced into the abdominal cavity and centered on the defect. Up to four sutures may be placed on the mesh’s cardinal points before its introduction and taken out transparietally using a suture passer for easier placement and fixation.

The final step of the procedure is mesh fixation: the mesh is fixed with non-absorbable tackers positioned along the cardinal axes and in a double crown conformation, the first along the edges, the second 2–3 cm from the edges. Mesh implantation may be further reinforced with absorbable tackers. Near bony prominences and areas at high risk for vascular lesions or nerve entrapment (e.g., near the triangle of pain in the inguinal region), the mesh can be fixed with cyanoacrylate glue.

## Results

Patients’ demographics are shown in Table 1. No differences existed between the groups regarding age and prevalence of smokers. OC1 had more female patients than OC2 (44 versus 22%, respectively). OC3 had more ASA score III patients than OC1 (36 versus 15%, respectively). OC2 and OC3 patients had a higher rate of comorbidities (76 and 81%, respectively, versus 64% in OC1), especially hypertension and cardiovascular diseases.

**Table 1:** Patients’ demographics

	OC1 (*n* = 231)	OC2 (*n* = 55)	OC3 (*n* = 36)	*p* value
**Age (years)**	62 (SD 11)	59 (SD 12)	59 (SD 11)	0.128
**Sex**				
Male	101 (44%)	12 (22%)	10 (28%)	0.004
Female	130 (56%)	43 (78%)	26 (72%)	*p* 1–2 0.007 *p* 1–3 0.154 *p* 2–3 0.831
**BMI (mean)**	32 (SD 1)	37 (SD 1)	44 (SD 5)	< 0.0001
**ASA score**				
II	197 (85%)	46 (84%)	23 (64%)	0.003
III	34 (15%)	9 (16%)	13 (36%)	*p* 1–3 < 0.001 *p* 2–3 0.093 *p* 1–2 0.365
**Smokers**	33 (14%)	14 (25%)	9 (25%)	0.057 *p* 1–2 0.105 *p* 1–3 0.255 *p* 2–3 0.993
**Comorbidities**	147 (64%)	42 (76%)	29 (81%)	0.042
Hypertension	113 (49%)	36 (65%)	22 (61%)	0.051
CV diseases	29 (13%)	14 (25%)	9 (25%)	0.02
COPD	21 (9%)	4 (7%)	7 (19%)	0.119
Diabetes	41 (18%)	12 (22%)	9 (25%)	0.473
Chronic liver disease	20 (9%)	7 (13%)	3 (8%)	0.632
Chronic kidney disease	2 (1%)	3 (5%)	0	0.034

Data are expressed as mean (*SD* standard deviation) or number (percentage)

*BMI* body mass index, *ASA* American Society of Anaesthesiologists, *CV* cardiovascular, *COPD* chronic obstructive pulmonary disease

Table 2 illustrates the characteristics of the abdominal wall defects. There were no differences among the groups regarding hernia type, mean defect size, mean defect area, and prevalence of giant hernias, multiple defects, swiss cheese defects and recurrent hernias. OC2 had more lateral defects than OC3 (24 vs 6%, respectively). More patients in OC1 had received previous operations than OC3 (93 vs 89%, respectively).

**Table 2:** Characteristics of the defect

	OC1 (*n* = 231)	OC2 (*n* = 55)	OC3 (*n* = 36)	*p* value
**Type of hernia**				
Primitive	33 (14%)	11 (20%)	8 (22%)	0.337
Incisional hernia	193 (84%)	41 (75%)	28 (78%)	0.257
Primitive + incisional	5 (2%)	3 (5%)	0	0.221
**Mean defect size (cm)**	7 × 10 (2 × 2–28 × 30)	7 × 10 (2 × 2–28 × 30)	7 × 9 (2 × 2–15 × 30)	
**Mean defect area (cm**^**2**^**)**	73 (3–628)	73 (3–396)	60 (3–220)	0.672
W1 (< 4 cm)	23 (10%)	6 (11%)	1 (3%)	0.518
W2 (4–10 cm)	120 (53%)	30 (56%)	23 (66%)	
W3 (> 10 cm)	85 (37%)	18 (33%)	11 (31%)	
**Giant hernias (> 15 cm)**	48 (21%)	11 (20%)	4 (11%)	0.395
**EHS classification**				
Midline defect	200 (87%)	42 (76%)	34 (94%)	0.162
Lateral defect	20 (9%)	9 (16%)	1 (3%)	
Midline + lateral defect	11 (5%)	4 (7%)	1 (3%)	
Presence of lateral defect	31 (13%)	13 (27%)	2 (6%)	0.043 *p* 1–2 0.125 *p* 1–3 0.418 *p* 2–3 0.042
**Multiple defects**	36 (16%)	11 (20%)	8 (22%)	0.505
**Swiss cheese defect**	38 (16%)	7 (12%)	6 (17%)	0.786
**Previous surgery**	214 (93%)	44 (80%)	32 (89%)	0.018 *p* 1–3 0.013 *p* 2–3 0.760 *p* 2–3 0.344
Multiple procedures	119/214 (56%)	19/44 (43%)	9/32 (28%)	
**Recurrent hernia**	55 (24%)	11 (20%)	8 (22%)	0.670
R I	49/55 (21%)	11/11 (20%)	4/8 (11%)	
R II	4/55 (2%)	0	3/8 (8%)	
R III	1/55 (0%)	0	1/8 (3%)	
**Previous hernia repair**				
Use of mesh	45/55	9/11	6/8	
Direct suture	10/55	2/11	2/8	

Data are expressed as mean (range) and number (percentage)

*EHS* European Hernia Classification, *R I* first recurrence, *R II* second recurrence, *R III* third recurrence

Intraoperative and 30-day postoperative variables are reported in Table 3. There were no differences in terms of intraoperative complications, conversions to open surgery, suture of the defect, use of transfascial sutures, number of associated procedures. Intraoperative complications were 9 (4%) in OC1 (four bleedings during lysis of adhesions, one full-thickness small bowel enterotomy treated with resection and anastomosis, two seromuscular tears of the small bowel and two seromuscular tears of the large bowel, all treated with suture), of which one bleeding and one small bowel enterotomy required a conversion to open approach. They were 3 (5%) in OC2 (one bleeding during lysis of adhesions, one full-thickness small bowel enterotomy treated with conversion to open approach followed by resection and anastomosis, one seromuscular tear of the small bowel treated with suture), and 1 (3%) in OC3 (one full-thickness small bowel enterotomy treated with suture). All enterotomies recognized intraoperatively resulted in minimal field contamination, and in no case this compromised mesh implantation.

**Table 3:** Intraoperative and 30-day postoperative variables

	OC1 (*n* = 231)	OC2 (*n* = 55)	OC3 (*n* = 36)	*p* value
**Operative time (min)**	137 (SD 76)	151 (SD 73)	144 (SD 68)	0.433
**Defect closure**	23 (10%)	8 (15%)	1 (3%)	0.186
**Transfascial sutures**	17 (7%)	7 (13%)	2 (6%)	0.355
**Use of more than one mesh**	5 (2%)	3 (5%)	0	0.221
**Mean mesh area** **(cm**^**2**^**)**	402 (SD 204)	386 (SD 189)	396 (SD 166)	0.861
**Mesh:defect area ratio**	12 (SD 10)	13 (SD 13)	12 (SD 8)	0.723
**Associated procedures**	35 (15%)	12 (20%)	7 (19%)	0.538
VLC	13 (6%)	6 (10%)	2 (5%)	
TAPP	12 (5%)	2 (3%)	3 (8%)	
Fundoplication	1 (0%)	0	0 (0%)	
Urological surgery	1 (0%)	0	0 (0%)	
Sleeve gastrectomy	0	1 (2%)	2 (5%)	
Other	8 (3%)	1 (2%)	0 (0%)	
**Intraoperative complications**	9 (4%)	3 (5%)	1 (3%)	0.801
**Conversion to open approach**	2 (1%)	1 (2%)	0	0.664
**Median length of stay (days)**	4 (IQT 3–5)	4 (IQT 3–5)	5 (IQT 3–9)	0.0006 *p* 1–2 0.815 *p* 1–3 0.0002 *p* 2–3 0.001
**At least one postoperative complication**	17 (7%)	3 (5%)	6 (17%)	0.120
Surgical site infection	2 (1%)	1 (2%)	0	
Surgical site hematoma	2 (1%)	0	1 (3%)	
Paralytic ileus	4 (2%)	1 (2%)	0	
Bowel perforation	0	0	1 (3%)	
Hematochezia	1 (1%)	0	0	
Cardiovascular complications	2 (1%)	0	2 (6%)	
Respiratory complications	5 (2%)	1 (2%)	1 (3%)	
Urinary complications	1 (1%)	0	1 (3%)	
Ischemic stroke	1 (1%)	0	0	
**Major complications (Clavien-Dindo > II)**	6 (2%)	1 (2%)	2 (6%)	0.449
**Clavien-Dindo grade**				
I	6 (2%)	1 (2%)	0	0.061
II	6 (2%)	0	4 (11%)	
IIIa	3 (1%)	0	0	
IVa	2 (1%)	1 (2%)	1 (3%)	
IVb	0	0	1 (3%)	
V	0	1 (2%)	0	0.090

Categorical variables are expressed as number (percentage)

Continuous data are expressed as mean (*SD* standard deviation) or median (*IQT* interquartile range), as appropriate

*VLC* videolaparoscopic cholecystectomy, *TAPP* TransAbdominal PrePeritoneal hernia repair, *SSI* surgical site infection

The overall rate of early postoperative complications was 26/322 (8%), with 9/322 (3%) major complications. OC3 had a longer median length of hospital stay (LOS) (5 days versus 4 days in the other groups, *p* = 0.0006).Postoperative complications and SSIs were similar between the groups. Major complications were two SSIs requiring negative-pressure wound treatment, one “missed” small bowel enterotomy requiring multiple reoperations, one ischemic stroke, two respiratory failures, one pulmonary embolism, a case of bilateral hydronephrosis requiring stent positioning, and a case of hematochezia requiring colonoscopy. OC2 had one in-hospital death due to pulmonary embolism.

**Fig. 1:**
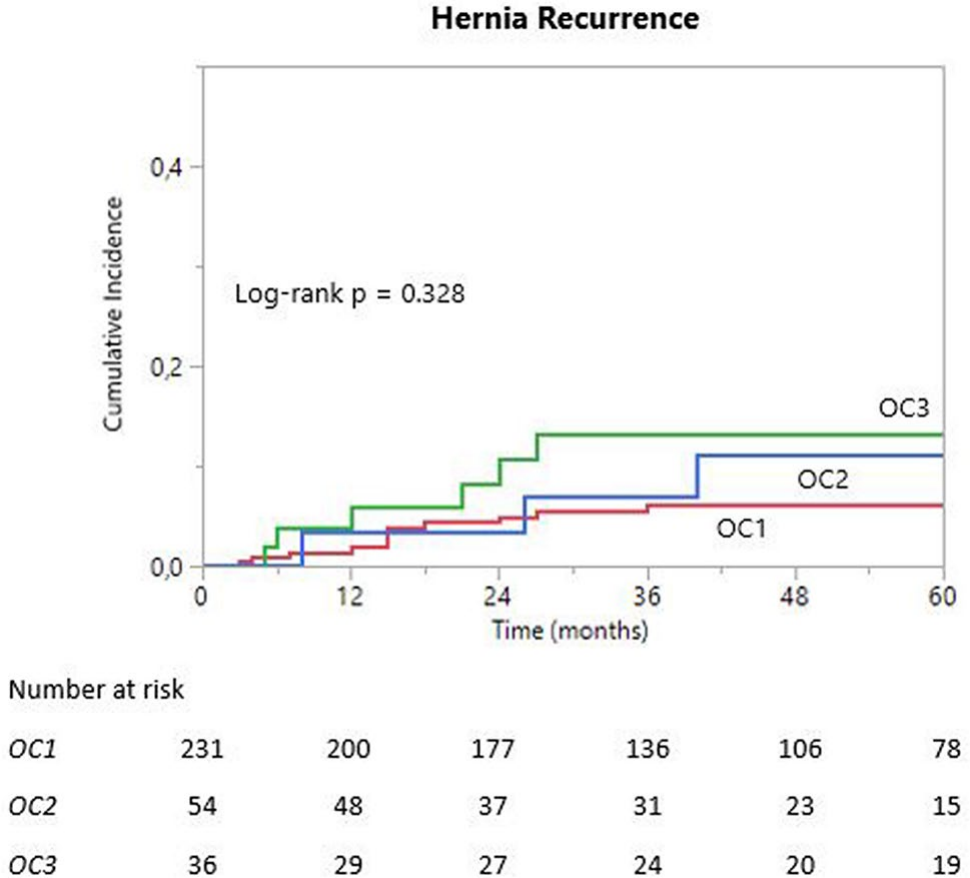
Kaplan Meier curve of hernia recurrence in Obesity Class I (OC1), II (OC2) and III (OC3) patients

Table 4 reports long-term outcomes. The groups had similar median follow up durations, with at least 76% patients having more than 2 years of follow up in all groups. Patients lost at follow up (i.e., follow up of 3 months or less) were 25 (19 OC1, 2 OC2, 4 OC3). There were no differences in seroma occurrence. Seromas requiring reoperation with mesh removal were 2 in OC1, 3 in CO2 and 2 in OC3. One patient in OC1 required reoperation with mesh removal due to bowel occlusion. Hernia recurrences across all groups were 22/321 (7%), of which 7 were reoperated (4 with LVHR, 3 with OVHR). The overall median time to hernia recurrence was 15 months, with an interquartile range of 7–26 months. There was no difference in median time to hernia recurrence between the three groups. 6/22 hernia recurrences occurred after 2 years (3 OC1, 1 OC2, 2 OC3), while only 1 OC1 patient had a recurrence after 5 years. The overall recurrence-free survival was 94% at 2 years (standard error, SE 0.05), 92% at 5 years (SE 0.08) and 91% at 10 years (SE 0.09). Kaplan Meier curves of the incidence of hernia recurrence in the three groups are shown in Fig. 1; there was no difference in recurrence rate groups (log-rank *p* = 0.328). The 2-year and 5-year recurrence-free survival were 95% (SE 0.05) and 94% (SE 0.06) for OC1; 89% (SE 0.11), and 87% (SE 0.13) for OC2; and 97% (SE 0.03) and 89% (SE 0.11) for OC3.

**Table 4:** Long-term variables

	OC1 (*n* = 231)	OC2 (*n* = 54)	OC3 (*n* = 36)	*p* value
**Median follow up (months)**	47 (27–81)	46 (24–73)	60 (26–108)	0.453
**Seromas**	55 (24%)	14 (26%)	8 (22%)	0.915
Type I–II	41/55	10/14	4/8	
Type III	5/55 (3 3a, 2 3d)	2/14 (2 3a)	1/8 (3a)	
Type IV	9/55 (5 4a, 1 4b, 3 4e)	2/14 (1 4b, 1 4c)	3/8 (all 4e)	0.464
**Chronic pain**	11 (5%)	5 (9%)	3 (9%)	0.383
**Pseudorecurrence**	6 (3%)	1 (2%)	1 (3%)	0.945
**Port-site hernia**	7 (3%)	0	2 (6%)	0.272
**Reoperation with mesh removal**	4 (2%) (1 due to bowel occlusion, 2 to infected seroma, 1 to persistent seroma)	3 (6%) (2 due to infected seroma, 1 to persistent seroma)	2 (6%) (both due to infected seroma)	0.176
**Hernia recurrence**	13 (6%)	6 (11%)	3 (8%)	0.328
Recurrence at 1-year po	2 (1%)	1 (2%)	1 (3%)	
Surgical repair	4 (2%) (2 laparoscopic, 2 open repair)	3 (6%) (2 laparoscopic, 1 open repair)	0	0.142

Data are expressed as median (interquartile range) and number (percentage)

*PO* postoperative, Seromas are defined according to the Morales-Conde classification [32]

There were no differences in recurrence, port-site hernia, pseudorecurrence or chronic pain between the three groups. One patient in OC1 was reoperated due to chronic pain with partial tacker removal.

Results of the univariate and multivariate analyses are shown in Table 5. Factors associated with 30-day postoperative complications at univariate analysis were COPD, ASA class III and smoking; OC3 and the presence of a giant hernia were weakly associated with the occurrence of complications (*p* = 0.06). At multivariate analysis, the only factor significantly associated with 30-day postoperative complications was COPD, with an OR of 3.08. Risk factors independently associated with recurrence were previous hernia repair and presence of a lateral defect, with an OR of 1.89 and 1.79, respectively. Regarding the occurrence of seroma, risk factors were immunosuppressive therapy, presence of a giant defect and more than one previous hernia repair (OR 5.34, 1.45 and 2.05, respectively).

**Table 5:** Univariate and multivariate analyses

	Univariate analysis		Multivariate analysis	
	OR (95% CI)	*p* value	OR (95% CI)	*p* value
**Recurrence**				
Previous hernia repair	3.82 (1.58–9.23)	0.003	1.89 (1.2–2.94)	0.006
Lateral defect	3.20 (1.23–8.38)	0.022	1.79 (1.08–2.88)	0.032
Male sex	2.52 (1.04–6.08)	0.042		ns
**Seroma**				
Immunosuppressive therapy	22.97 (1.17–449.8)	0.013	5.34 (1.6–62)	0.003
Giant defect (> 15 cm)	1.98 (1.09–3.60)	0.032	1.45 (1.07–1.95)	0.018
More than one previous hernia repair	4.17 (1.09–15.92)	0.039	2.05 (1.07–4.06)	0.035
Swiss cheese defect	1.95 (1.03–3.71)	0.049		ns
**30-day postoperative complications**				
COPD	9.6 (3.9–23.5)	< 0.0001	3.08 (1.97–4.79)	< 0.0001
ASA III	4.09 (1.76–9.48)	0.002		ns
Smoking	2.8 (1.17–6.64)	0.027		ns
Obesity class III	2.66 (0.99–7.13)	0.055		ns
Giant defect (> 15 cm)	2.37 (1–5.6)	0.067		ns

*OR* odds ratio, *CI* confidence interval, *COPD* chronic obstructive pulmonary disease,* ASA* American Society of Anaesthesiologists, *ns* non-significant

## Discussion

Our study shows that OC3 was associated with longer median LOS, but without differences in postoperative complications and long-term outcomes compared with OC1 and OC2, further proving that LVHR is safe and effective also in patients with morbid obesity.

LVHR in patients with obesity is considered a more challenging procedure than in nonobese patients [5]. Obesity is often associated with large defects [19] and multiple defects which may have been clinically misrecognized. In addition, the same mechanisms that make patients with obesity more subject to development of VH than the general population may be implied in recurrence. For these reasons, LVHR in obese patients may require additional steps, such as a “plus” technique with closure of the defect, and implantation of larger meshes that guarantee a wider overlap [10].

The primary outcome of this study was hernia recurrence. Reports of recurrences after LVHR in the obese population vary between 4 and 21% [20]. In our series, the overall recurrence rate was 7% (22/321) after a median follow up of 49 months, with an overall recurrence-free survival of 91% at 10 years. Although not significant, OC2 had a trend toward a greater recurrence rate (11 versus 6% in OC1 and 8% in OC3). This may be partially explained by the higher presence of lateral defects in OC2 (27 versus 13% in OC1 and 6% in OC3, *p* = 0.04). Indeed, the presence of a lateral defect was associated with recurrence at multivariate analysis, together with previous hernia repair. Non-midline incisional hernias have been demonstrated to have higher recurrence rate, especially when combined with obesity [19], and both lateral hernia and previous mesh repair are included in the criteria for the definition of a complex abdominal wall hernia [21]. A thorough preoperative evaluation is essential to identify patient-specific risk factors and defects located in unfavorable positions, so as to offer the most appropriate treatment strategy for each patient.

In our series, the range of defect sizes was broad in all groups, with similar distributions of W1, W2 and W3 sized defects. The 2019 update of the International Endohernia Society (IEHS) guidelines for LVHR [5] recommends that laparoscopic repair should be limited to hernias < 15 cm, due to the higher recurrence rate and postoperative complications; in our series, giant hernias were not associated with increased recurrence, but they were associated with a higher incidence of seromas and weakly associated with early postoperative complications at univariate analysis.

The mean mesh:defect area ratio (MDAR) was of 13, which has been shown to be an acceptable ratio to prevent recurrence [22]. Eight cases required the use of two meshes to ensure the proper overlap. The larger overlap given by a larger mesh size, or even two meshes, has to be weighted against the potentially increased risk of complications due to the higher quantity of implanted foreign material, such as seromas and visceral adhesions, and of means of fixations, especially when in close proximity to the abdominal wall borders, a risk factor for chronic pain and hernia recurrence [23, 24]. In our experience, implantation of more than one mesh was not associated with worsened outcomes, and can be considered when adequate overlap can be safely reached laparoscopically and without fixing the mesh too close to the borders; if those conditions can’t be guaranteed, another kind of approach should be preferred.

Intraoperative variables were similar between the three groups, with no differences in conversion rate, intraoperative complications, or mean operative time. The 13 intraoperative complications that occurred in our series were either bleeding or enterotomies, and the majority of them (10/13, 77%) could be managed laparoscopically. This vouches for the feasibility of the procedure also in the case of increasing BMI, and confirms the results obtained from previous studies [7, 15, 25].

The impact of increasing BMI on postoperative complications was recently investigated by Owei et al. [26], who stratified 55′180 patients undergoing minimally invasive VHR into seven BMI classes (from < 18.5 to ≥ 50). They found an increase in complication rates with increasing BMI, with BMI ≥ 50 as an independent risk factor for surgical and medical postoperative complications. In our series, there were no differences in complications between the groups, however OC3 had a trend toward a greater complication rate, concerning both overall complications (17 versus 7% in OC1 and 5% in OC3, respectively) and major complications (6 versus 2% in OC1 and OC2). OC3 had more complex patients, with a higher number of ASA III patients, more smokers and more patients with comorbidities. This higher complexity may justify the longer median LOS in OC3 patients (5 days versus 4 in the other groups, *p* = 0.0006) and the trend toward an increased incidence of complications. Indeed, smoking habit and ASA III are known risk factors for surgical site infections [27] which, while not statistically significant, were higher in OC3 than in the other groups (8 versus 2% in the other groups). Preoperative optimization of modifiable risk factors, such as smoking cessation and weight loss, should be implemented whenever possible, especially in patients with morbid obesity.

At multivariate analysis, COPD was the only independent risk factor for postoperative complications. This association has already been observed [28, 29], both in abdominal procedures in general and in VHR in particular. COPD is especially associated with respiratory complications, which are frequent complications following VHR, likely due to the increase in intra-abdominal pressure due to the hernia sac reduction with the consequent decrease in pulmonary compliance [30]. Laparoscopy has long been known to be associated with better postoperative respiratory function than open surgery [31], thus should be the preferred approach in COPD patients even if they have a higher risk of complications than the general population.

Reports of seroma occurrence following LVHR vary between 0.5 and 78% [32], although its incidence may be underreported when no postoperative radiological evaluation is conducted, as many seromas are asymptomatic and not clinically detectable. In our series, overall seroma incidence was 24%, while incidence of major seroma-related complications (Morales-Conde type IV) was 4%. While not significant, OC3 had a tendency to develop more serious seromas (3/8 OC3 seromas were type IV, versus 9/55 OC1 and 2/14 OC2). Our relatively high incidence of seromas may be related both to the mesh type (a study by Susmallian et al. [33] has shown a 100% radiological evidence of seroma occurrence after LVHR with an ePTFE mesh), and to the high rate of W3 and giant defects and the consequent low rate of defect closures, which are risk factors for seroma development [34]. In our experience, factors significantly associated with seroma occurrence at multivariate analysis were presence of a giant defect, more than one previous hernia repair and immunosuppressive therapy. The role of immunosuppressive therapy as a risk factor for SSO has recently been investigated by Haskins et al. [35] in a study including 3537 patients who underwent VHL: they concluded that immunosuppressed patients had more SSO than the control group, with seromas being the most common SSO. Our study confirms these results.

This study has some limitations. It’s an observational retrospective study of a monocentric experience. The three groups have different sample sizes, which may influence their comparability, especially considering the small sample size of OC3. The study considers a time period of a decade, during which changes in clinical practice and technical advances may have influenced the outcomes. This however allows for a median follow up of almost four years in all groups.

Strengths of the study are the standardized surgical technique and the use of a single mesh type in all patients, with the same means of fixation.

## Conclusion

Our study on  patients with obesity shows that LVHR is a safe and effective treatment for VH even in case of increasing BMI. Indeed, increasing BMI did not lead to an increased rate of postoperative complications, nor it worsened long-term outcomes. Morbid obesity was only associated with a one-day median increase in LOS. Risk factors for recurrence were not linked to patient characteristics, but to the presence of a lateral defect and to a previous hernia repair, suggesting that the choice of the approach should take into account the characteristics of the defect more than the BMI of the patient.
